# Influences of carbon concentration on crystal structures and ideal strengths of B_2_C_x_O compounds in the B-C-O system

**DOI:** 10.1038/srep15481

**Published:** 2015-10-21

**Authors:** Meiguang Zhang, Haiyan Yan, Baobing Zheng, Qun Wei

**Affiliations:** 1 College of Physics and Optoelectronic Technology, Nonlinear Research Institute, Baoji University of Arts and Sciences, Baoji 721016, China; 2 College of Chemistry and Chemical Engineering, Baoji University of Arts and Sciences, Baoji 721013, China; 3 School of Physics and Optoelectronic Engineering, Xidian University, Xi’an 710071, China

## Abstract

The search for novel superhard materials with special structures and improved thermal stability and hardness remains considerably experimental and theoretical challenges. Recent reports proposed that higher carbon content in ternary B_2_C_x_O compounds, which are isoelectronic with diamond, would lead to increased strength and hardness. This notion was derived from the calculated elastic parameters and empirical hardness formulas based on structural and electronic properties of the equilibrium structures. In present work, we introduce three potential ultra-incompressible and thermodynamically stable B_2_C_x_O (*x* ≥ 2) phases via a systematic particle swarm optimization algorithm structure searches. By evaluating the trends of the crystal configuration, electronic structure, and mechanical properties as a function of the C concentration, it is found that the high carbon concentration benefits the formation of the *sp*^3^ C-C covalent bonds and leads to the enhanced elastic moduli and ideal strengths in these B_2_C_x_O compounds. Studies of strain-stress behavior at large deformation, however, indicate that all these B_2_C_x_O compounds possess substantially lower ideal shear strengths than those of diamond and *c*-BN, suggesting that they may not be intrinsically superhard.

The quest for intrinsic superhard materials is inspired by scientific curiosity and the need of materials with high hardness, high thermal stability, and oxidation resistance in industrial applications. Since successful laboratory syntheses of diamond and cubic boron nitride (*c*-BN), efforts to synthesize other novel materials with improved thermal stability and hardness have been actively pursued^1^,^2^,^3^. Traditionally, it is commonly accepted that superhard materials are formed by light elements (B, C, N and O) for the belief that relatively short bond lengths and strong covalent bonds in these materials, which make them to be primary candidates for low-compressibility or superhard materials. Following this strategy, significant progresses have been achieved in synthesizing superhard BC_x_N^4^,^5^,^6^,^7^,^8^,^9^, BC_x_^10^,^11^, *γ*-B_28_^12^, and B_x_O^13^,^14^,^15^ which has opened up a new route to research superhard materials in B-C-N-O systems. Of particular interest is the synthesized ternary superhard material, BC_2_N, the second hardest known material with a Vickers hardness of 76 GPa^5^. Meanwhile, there are also several theoretical approaches^16^,^17^,^18^ for designing and predicting new potential superhard materials to complement experimental works. A characteristic feature of the electronic structure of diamond, essential for its hardness, is the presence, in the valence level of each carbon atom, of four electrons capable of taking part in the formation of four strong tetrahedrally directed covalent bonds. Thus in the search for new superhard materials, it is natural to try to reproduce this property of diamond. The composition of a multicomponent light elements compound isoelectronic with diamond involves the determination of a set of solutions of indefinite equations of the form^16^.
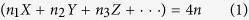
where *n*_1_, *n*_2_, *n*_3_ · · · are the numbers of valence electrons in the atoms of the components, *n* is an integral positive number, and *X*, *Y*, *Z* · · · are the required concentrations.

In contrast to the extensively studied B-C-N system, the ternary B-C-O compounds have become the subject of close attention during the last decade. Experimentally, several B-C-O materials at high pressure and high temperature have been synthesized, such as single crystals of interstitial phases B(C,O)_x_^19^,^20^. Recently, a potential superhard ternary material B_2_CO which is isoelectronic with diamond, stabilized in two tetragonal diamond-like structures (tP4 and tI16) with claimed hardness of 50 GPa was predicted by Li *et al*.^21^. Moreover, it was also proposed that more ternary compounds which are isoelectronic with diamond exist in B-C-O system, such as B_2_C_x_O (*x* ≥ 2) with higher carbon content could possess higher strength and hardness than those of B_2_CO. This is based on the intuitive argument that more carbon content should bring the hybrid compounds closer to diamond in their mechanical properties because of the larger proportion of the strong *sp*^3^ C-C bonds in the structures. It was more recently proposed that an orthorhombic B_2_C_5_O phase within *Cmm*2 symmetry^22^ exhibits higher hardness (66.1 GPa) than that of B_2_CO. These important pioneering works give us broadened views into the essence of atomic binding in solids while inevitably advance our understanding on the structural stability and superior properties of B_2_C_x_O in the B-C-O system. Therefore, to extend the ultrahard structures for other family members and investigate the effects of the carbon concentration on the mechanical behaviors of B_2_C_x_O (*x* ≥ 2) compounds are of great interest and highly desirable. To address these issues, we here perform extensive structure searches to explore the potential energetically stable B_2_C_x_O (*x* ≥ 2) phases at ambient pressure using the prevalent developed Crystal structure AnaLYsis by Particle Swarm Optimization package (CALYPSO)^23^,^24^, unbiased by any known information. This method has been successfully applied to extensive structures which have been confirmed by independent experiments^25^,^26^,^27^,^28^,^29^. Two typical examples: the experimental crystal structures of cold-compressed graphite phase and synthesized cubic BC_3_ phase which have been recently resolved^25^,^29^. Three novel tetragonal diamond-like structures with group symmetry *I*4_1_/*amd*, *I*-4*m*2, and *P*-4*m*2 for B_2_C_2_O, B_2_C_3_O, and B_2_C_5_O were uncovered, surprisingly, all these new structures can be derived from the previous proposed tP4-B_2_CO supercells. First principles calculations were then executed to characterize the trends of the crystal structures, electronic structures, and mechanical behaviors of these predicted phases.

## Results and Discussion

**Figure 1:**
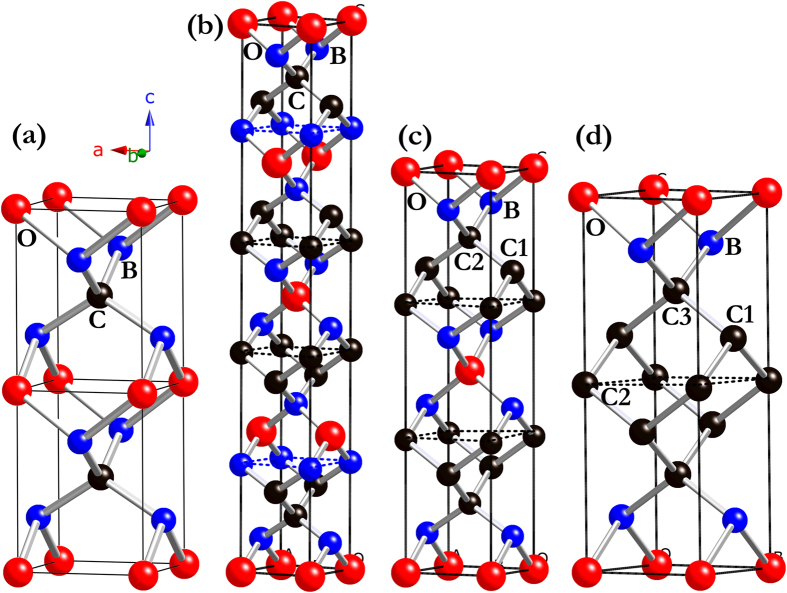
Crystal structures of B_2_C_x_O. (**a**) 1 × 1 × 2 supercell of tP4-B_2_CO; (**b**) *I*4_1_/*amd-*B_2_C_2_O, B atoms occupy the 8*e* (0, 0, 0.3044), C atoms occupy the 8*e* (0, 0, 0.1002), and O atoms occupy the 4*b* (0, 0, 0.5) positions; (**c**) *I*-4*m*2-B_2_C_3_O, B atoms occupy the 4*f* (0, 0.5, 0.5910), C1 atoms occupy the 2*c* (0, 0.5, 0.25), C2 atoms occupy the 4*e* (0, 0, 0.3317), and O atoms occupy the 2*a* (0, 0, 0) positions; (**d**) *P*-4*m*2-B_2_C_5_O, B atoms occupy the 2 *g* (0, 0.5, 0.8624), C1 atoms occupy the 2 *g* (0, 0.5, 0.3783), C2 atoms occupy the 1*d* (0, 0, 0.5), C3 atoms occupy the 2*f* (0.5, 0.5, 0.2551), and O atoms occupy the 1*a* (0, 0, 0) positions. The blue, black, and red spheres represent B, C, and O atoms, respectively.

Variable-cell simulations with 1–4 formula units (f.u.) in the unit cell were performed at ambient pressure, and our structure searches uncover the most stable structure for B_2_C_2_O, B_2_C_3_O, and B_2_C_5_O to be tetragonal *I*4_1_/*amd* (4 f.u./cell), *I*-4*m*2 (2 f.u./cell), and *P*-4*m*2 (1 f.u./cell) phase, respectively. All these three structures are schematically showed in Fig. 1, together with the previous proposed tP4-B_2_CO (2 f.u./cell, space group: *P*-4*m*2)^21^. As presented in Fig. 1, the structure characteristics of B_2_C_x_O compounds with increasing carbon content can be revealed as follows. Firstly, there exists a striking structural connections between B_2_C_x_O (*x* = 2, 3, 5) and tP4-B_2_CO, that each B_2_C_x_O compound can be derived from a different tP4-B_2_CO supercell with replacement of the partial O and B atoms by C atoms. For example, the predicted *P*-4*m*2-B_2_C_5_O (Fig. 1(d)) can be constructed by a 1 × 1 × 2 tP4-B_2_CO supercell (Fig. 1(a)) by replacing two boron atoms at 2*g* and one oxygen atom at 1*c* positions to ensure the stoichiometry of B:C:O is 2:5:1. Secondly, similar to the atomic bonding behaviors in tP4-B_2_CO, all nonequivalent atoms in each B_2_C_x_O compound are all tetrahedrally bonded with *sp*^3^ bonding environment. Moreover, in Figures of 1(b)-1(d) for B_2_C_2_O, B_2_C_3_O, and B_2_C_5_O, the average number of C-C bond in their building blocks (which can be viewed as a pseudo-tetragonal tP4-B_2_CO unit cell, see the dashed cell in Fig. 1) increases from 1.6 to 4 with the increasing of carbon content. Thirdly, compare to those of in B_2_CO and B_2_C_2_O, the increasement of *sp*^3^ C-C tetrahedral covalent bonds can be clearly revealed in B_2_C_3_O and B_2_C_5_O at elevated C concentrations, as shown in Fig. 1(c,d). Therefore, high carbon content might benefits the mechanical properties of these B_2_C_x_O compounds. The dynamical stability of a crystalline structure requires the eigen frequencies of its lattice vibrations be real for all wavevectors in the whole Brillouin zone. We have thus performed the phonon dispersion calculations for *I*4_1_/*amd-*B_2_C_2_O, *I*-4*m*2-B_2_C_3_O, and *P*-4*m*2-B_2_C_5_O at ambient pressure, as presented in Figure S1. Clearly, no imaginary phonon frequency was detected in the whole Brillouin zone for each predicted tetragonal phase, indicating their dynamical stabilities at ambient conditions. However, the earlier proposed *Cmm*2-B_2_C_5_O^22^ is dynamical unstable with a wide range of imaginary frequency in the Brillouin zone according to our phonon dispersion calculations. The orthorhombic *Cmm*2 phase thus can be excluded from the candidates of B_2_C_5_O.

The calculated equilibrium lattice constants, average atomic volumes, bulk moduli, and pressure derivatives of bulk moduli for B_2_C_x_O compounds are listed in Table 1, along with previous theoretical values of tP4-B_2_CO^21^ for comparisons. It can be seen that the lattice parameter *a* of each B_2_C_x_O (*x* = 2, 3, 5) is close to that of tP4-B_2_CO, and the lattice parameter *c* of each B_2_C_x_O phase is about five, three, and two times that of tP4-B_2_CO, respectively. This is in agreement with crystal configuration of each B_2_C_x_O pahse as presented in Fig. 1. The bulk moduli and their pressure derivatives are obtained by fitting pressures and volumes with the third-order Birch-Murnaghan equation of state (EOS)^30^. More detailed structural information on the interatomic distances of these predicted phases are presented in Supplementary Table S1. From Table 1, one can see that the bulk moduli and densities of B_2_C_x_O compounds increase continuously with high carbon content, agree well with the decreasement of interatomic distances (see Table S1) in B_2_C_x_O compounds. Note that the *sp*^3^ C-C tetrahedral covalent bond lengths of B_2_C_3_O (1.585 Å) and B_2_C_5_O (1.575 and 1.568 Å) are slightly longer than that of diamond (1.535 Å), indicating they might possess excellent mechanical properties. Furthermore, it is important to explore the thermodynamic stability for further experimental synthesis. The thermodynamic stability for each B_2_C_x_O compound, with respect to the separate phases as a function of pressure, is quantified in terms of the formation enthalpies in two possible routes:
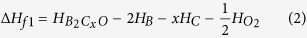
and
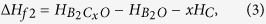
where the *α*-B^31^, diamond C, *α*-O_2_^32^, and *I*4_1_/*amd*-B_2_O^21^ are chosen as the reference phases. As shown in Figure S2, the calculated formation enthalpies obtained by this two reaction routes are all negative, which indicate that the formations of these three phases are exothermic at ambient as well as at high pressures. Thus these B_2_C_x_O compounds are stable against the decomposition into the mixture of B + C + O or B_2_O + C and the syntheses of these structures are highly desirable at very readily attainable pressures.

**Figure 2:**
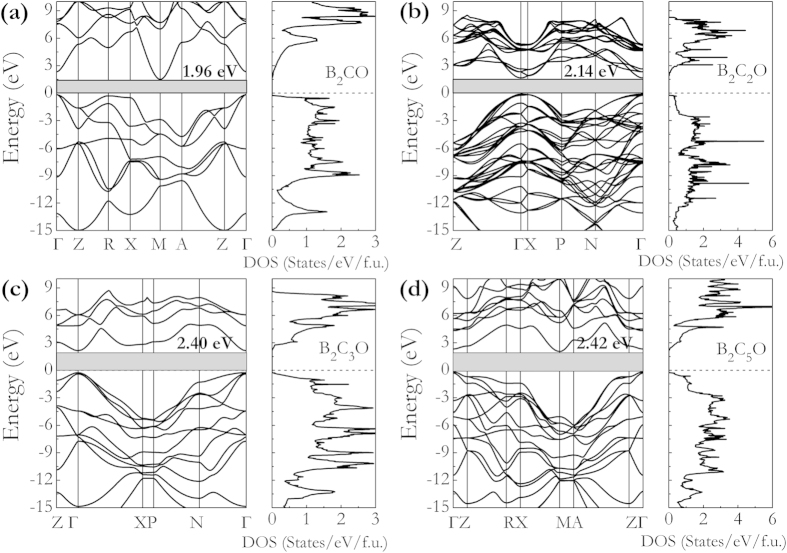
Calculated band structures and total DOSs of B_2_C_x_O at ambient pressure. The dashed horizontal line is Fermi energy.

**Figure 3:**
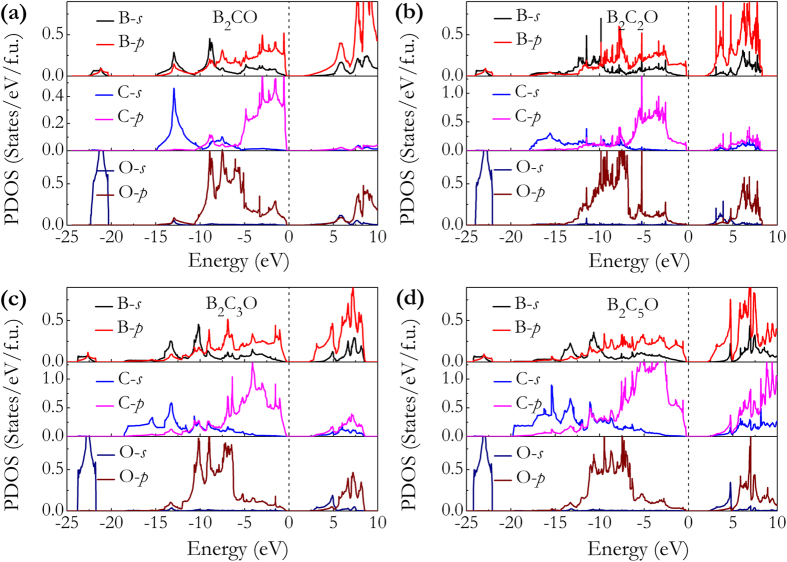
Calculated partial DOSs of B_2_C_x_O at ambient pressure. The dashed vertical curve is Fermi energy.

Figure 2 and 3 show the band structures, total density of states (DOS), and partial DOS for these B_2_C_x_O compounds. As shown in Fig. 2(a–d), the indirect semiconductor nature of B_2_CO and B_2_C_5_O are characterized by energy gaps of 1.96 and 2.42 eV between M and Γ points, while B_2_C_2_O and B_2_C_3_O are direct semiconductors with band gaps of 2.14 and 2.40 eV at Γ points in Fig. 2 (b,c). The increasement of band gaps of these B_2_C_x_O compounds can be clearly disclosed with increasing carbon contents. From inspection of their partial DOS curves in Fig. 3, it shows that the peaks from −24 to −21 eV have predominantly O-2*s* character. From Fig. 3(a–d), the weight of the C-2*s* and C-2*p* states increases gradually and the increased overlap of C-2*s* and C-2*p* in energy of −19 to −7 eV (B-2*p* and C-2*p* in energy of −7 eV to 0 eV ) leads to an enhanced hybridization interactions between C-C atoms (B-C atoms). To analyze the chemical bonding character of different atoms in these B_2_C_x_O compounds, electron localization function (ELF)^33^ and Bader charge analyses^34^ are calculated as presented in Figure S3 and listed in Table 2. For the selected 3D ELF distributions (ELF = 0.9) of B_2_C_3_O and B_2_C_5_O, the high electron localization can be seen in the regions of C-C bond and B-C bond, which is indicative of strong covalent bonding. However, at the same ELF value for B-O bond, the ELF attains local maximum at the O sites compared to B sites, reflecting the ionicity of B-O bond. From Bader charge analysis results listed in Table 2, the strong covalent nature of the C-C and B-C bonds in B_2_C_x_O were quantitatively revealed by the evidences of large charge densities at their bond critical points with negative Laplacian values. Especially for B_2_C_5_O, the charge density at C1-C2 bond critical point is 1.549 electrons/Å^3^ with a Laplacian value of −11.068, which is close to that of C-C bond in diamond^35^. However, compared to C-C and B-C bonds, the charge densities at B-O bond critical points are much smaller (0.678 ~ 0.703 electrons/Å^3^) and the corresponding Laplacian values are all positive, suggesting the intrinsic ionic bonding of B-O bonds in these compounds. We further found that the B → O charge transfer decreases from 1.533 *e* to 1.513 *e* with C concentration, resulting in weakened ionicity in B-O bond.

For the tetragonal B_2_C_x_O phase, six independent elastic constants *C*_*ij*_ were determined at GGA level^36^ from the stress of the strained structure with a small finite strain. The elastic stability, incompressibility, and rigidity of each tetragonal B_2_C_x_O compound are thus studied based on the calculated elastic constants and derived Hill elastic moduli^37^, as listed in Table 3. Firstly, one can see that the mechanical stabilities of three B_2_C_x_O compounds satisfy the Born-Huang criterion for a tetragonal crystal^38^ [*C*_11_ > 0, *C*_33_ > 0, *C*_44_ > 0, *C*_66_ > 0, *C*_11_ − *C*_12_ > 0, *C*_11_ + *C*_33_ − 2*C*_13_ > 0, 2(*C*_11_ + *C*_12_) + *C*_33_ + 4*C*_13_ > 0], indicating that they are mechanically stable at ambient pressure. For B_2_C_5_O, we find its unusually high incompressibility along *a*-direction, as demonstrated by the extremely large *C*_11_ value (889 GPa) which is slightly larger than experimental data of *c*-BN (*C*_11_: 820 GPa)^39^ and is comparable to that of known superhard diamond (*C*_11_: 1076 GPa)^40^. Secondly, our calculated elastic parameters for tP4-B_2_CO are in a good accordant with the previous theoretical results^21^ using the same exchange-correlation functionals: local density approximation (LDA)^41^. Moreover, the calculated Hill bulk moduli agree well with those directly obtained from the fitting of the Birch-Murnaghan EOS (see Table 1), which further demonstrates the accuracy of our elastic constants calculations for three B_2_C_x_O compounds. Thirdly, a parallel increasement of elastic moduli and a reduction of Poisson’s ratios can be clearly revealed with increasing carbon contents, as shown in Table 3. Since the hardness is deduced from the size of the indentation after deformation, a hard material typically requires a high bulk modulus to support the volume decrease created by the applied pressure, and a low Poisson’s ratio (*ν*) or high shear modulus (so that the material will not deform in a direction different from the applied load)^42^. Among these ternary compounds, the calculated bulk modulus for B_2_C_3_O and B_2_C_5_O is 322 GPa and 345 GPa, respectively, indicating their highly incompressible nature. According to Teter^43^, the shear modulus is a significantly better qualitative predictor of hardness than the bulk modulus, governing the indentation hardness. The shear moduli of B_2_C_3_O and B_2_C_5_O are 302 and 351 GPa, and they are expected to withstand shear strain to a large extent. In view of the large bulk and shear moduli of these B_2_C_x_O compounds, the hardness calculations are of great interest. By using three different hardness models proposed by Gao *et al*.^44^, Chen *et al*.^45^, and Šimůnek *et al*.^46^, the theoretical hardness of these B_2_C_x_O phases were estimated and listed in Table 4. One can see that our calculated theoretical hardness for tP4-B_2_CO is in excellent with previous result performed by Li *et al*.^21^ using the Gao’s model. For other new predicted B_2_C_2_O, B_2_C_3_O, and B_2_C_5_O phase, the calculated theoretical hardness is in the range of 43~57GPa, 47~62GPa, and 51~68 GPa, respectively, suggesting their potential superhard nature.

**Figure 4:**
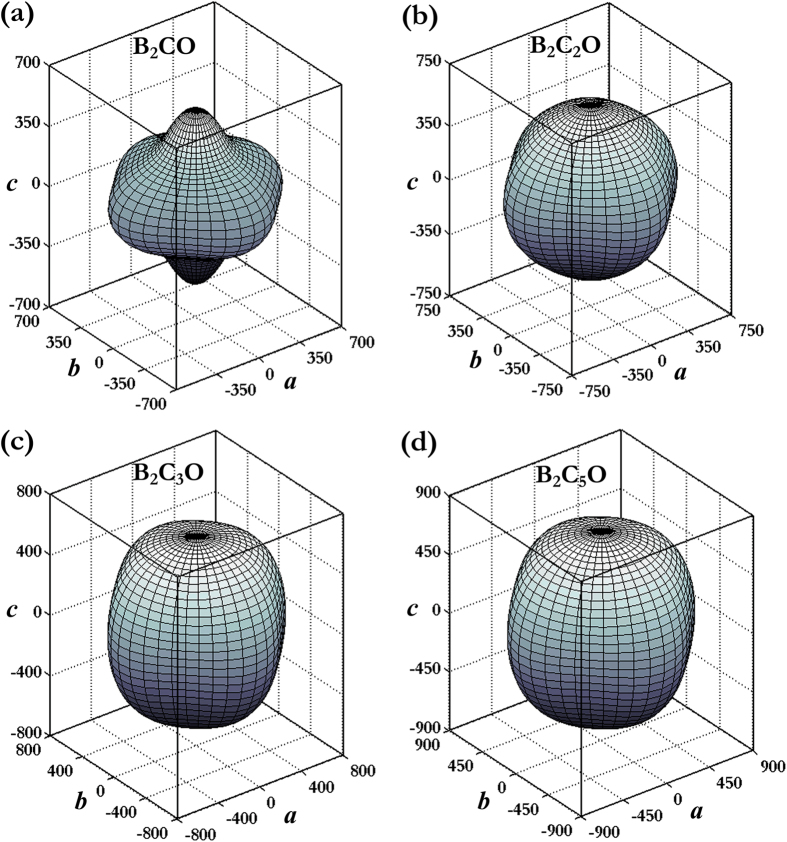
Three-dimensional surface representations of the Young’s Modulus *E*. (**a**) tP4-B_2_CO, (**b**) B_2_C_2_O, (**c**) B_2_C_3_O, and (**d**) B_2_C_5_O.

**Figure 5:**
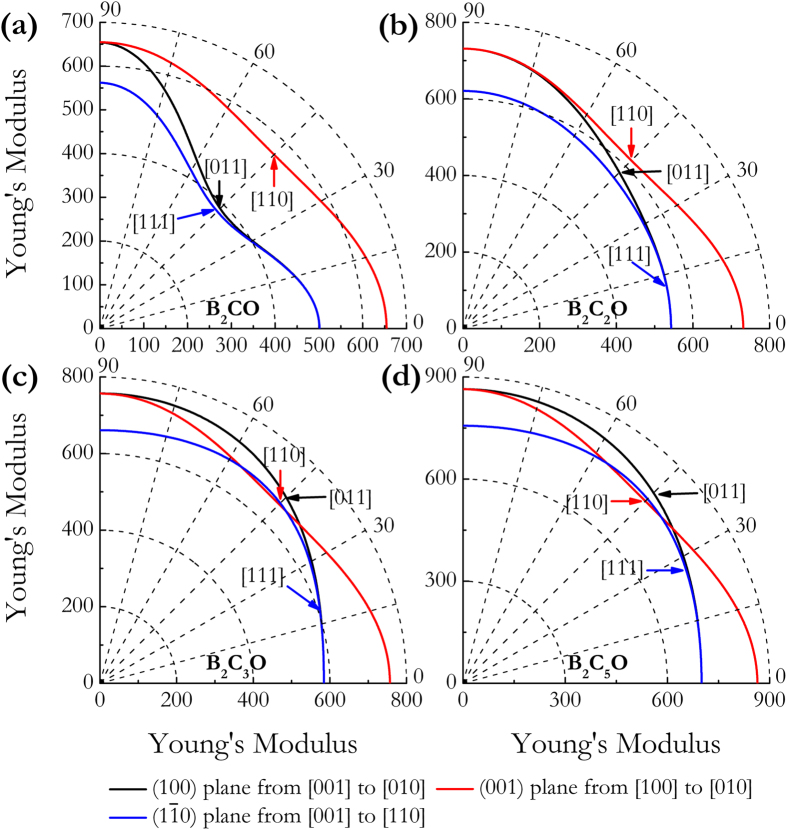
Orientation dependences of the Young’s modulus *E*. (**a**) tP4-B_2_CO, (**b**) B_2_C_2_O, (**c**) B_2_C_3_O, and (**d**) B_2_C_5_O.

**Figure 6:**
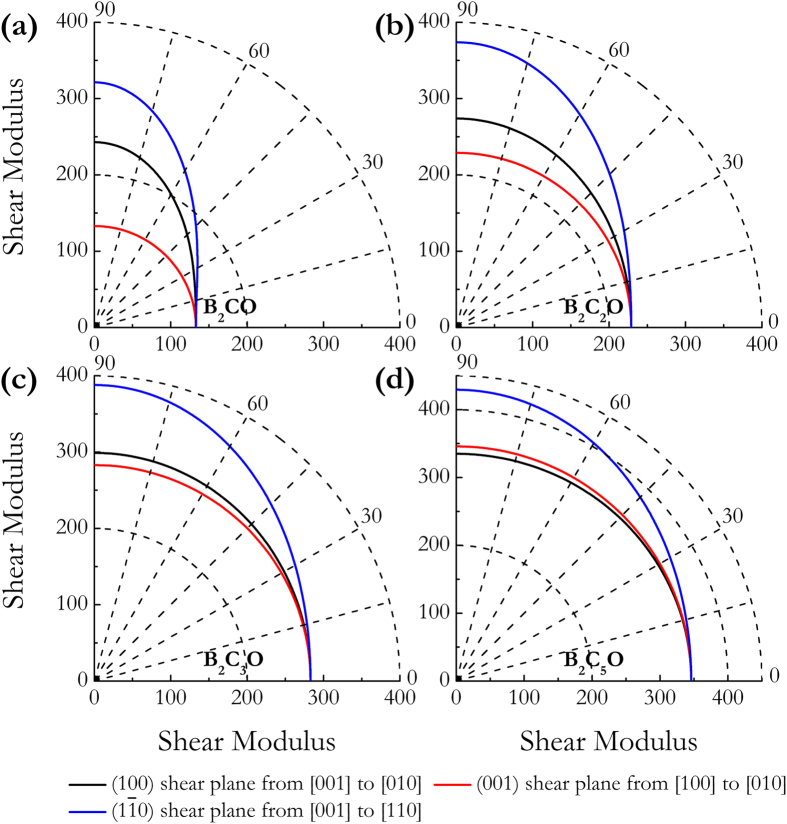
Orientation dependences of the Shear modulus *G*. (**a**) tP4-B_2_CO, (**b**) B_2_C_2_O, (**c**) B_2_C_3_O, and (**d**) B_2_C_5_O.

The elastic anisotropy of crystal can exert great effects on the properties of physical mechanism, such as anisotropic plastic deformation, crack behavior, and elastic instability. Therefore, the elastic anisotropies of tetragonal B_2_C_x_O compounds were systematically studied for their further engineering applications. We here calculated the orientation dependences of the Young’s modulus *E* and shear modulus *G*. For each tetragonal B_2_C_x_O phase, the Young’s modulus *E* is described by the following equation^47^:
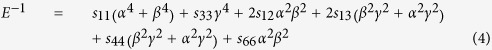
where *α*, *β*, and *γ* are the direction cosines which determine the angles between the *a*-, *b*-, and *c*-axis of a crystal and a given direction [*uvw*]. And *s*_11_, *s*_33_, *s*_44_, *s*_66_, *s*_12_, and *s*_13_ are the elastic compliance constants which are given by Kelly *et al*.^48^ Similarly, the orientation dependence of the shear modulus for arbitrary shear plane (*hkl*) and shear directions [*uvw*] is given by:
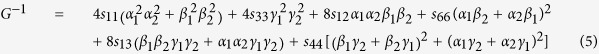
where (*α*_*1*_*, β*_*1*_*, γ*_*1*_) and (*α*_*2*_*, β*_*2*_*, γ*_*2*_) are the direction cosines of the [*uvw*] and [*HKL*] directions, and the [*HKL*] denotes the vector normal to the (*hkl*) shear plane. In Fig. 4, the distance from the origin of system of coordinate to this surface equals to the Young’s modulus in a given direction. For a perfectly isotropic medium this three-dimensional surface should be a sphere, however, all these B_2_C_x_O compounds exhibit a well-pronounced anisotropy in Fig. 4. In more detail, Fig. 5 presents the orientation dependences of Young’s modulus *E* along tensile axes within (001), (100), and 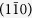 specific planes. One can see that (1): the maximum values of tP4-B_2_CO (655 GPa), B_2_C_2_O (732 GPa), B_2_C_3_O (757 GPa), and B_2_C_5_O (864 GPa) are all along the [100] directions, and the minimum value of tP4-B_2_CO (379 GPa), B_2_C_2_O (543 GPa), B_2_C_3_O (584 GPa), and B_2_C_5_O (734 GPa) is along [111], [111], [001], and [001] directions, respectively; (2) the value of *E*_*max*_/*E*_*min*_ for tP4-B_2_CO, B_2_C_2_O, B_2_C_3_O, and B_2_C_5_O is 1.73, 1.35, 1.30, and 1.18, respectively; and (3) the variations of the Young’s moduli for B_2_C_3_O and B_2_C_5_O along different directions decrease in the same following sequence: *E*_[100]_ > *E*_[011]_ > *E*_[110]_ > *E*_[111]_ > *E*_[001]_. As plotted in Fig. 6, the orientation dependence ofthe shear modulus *G* of each B_2_C_x_O compound was also calculated for shear on (001), (100), and 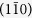 planes, respectively. Firstly, the shear moduli of the B_2_C_x_O compounds are all independent of the shear directions from [100] to [010] directions within their (001) basal plane, which is a result of the isotropy of elasticity in the basal plane for tetragonal crystal. Secondly, except for B_2_C_5_O, the smallest shear moduli for other B_2_C_x_O members are all distributed within (001) basal plane. Thirdly, compared to other B_2_C_x_O compounds, B_2_C_5_O possesses the lowest degree of anisotropy within (001), (100), and 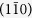 shear planes.

**Figure 7:**
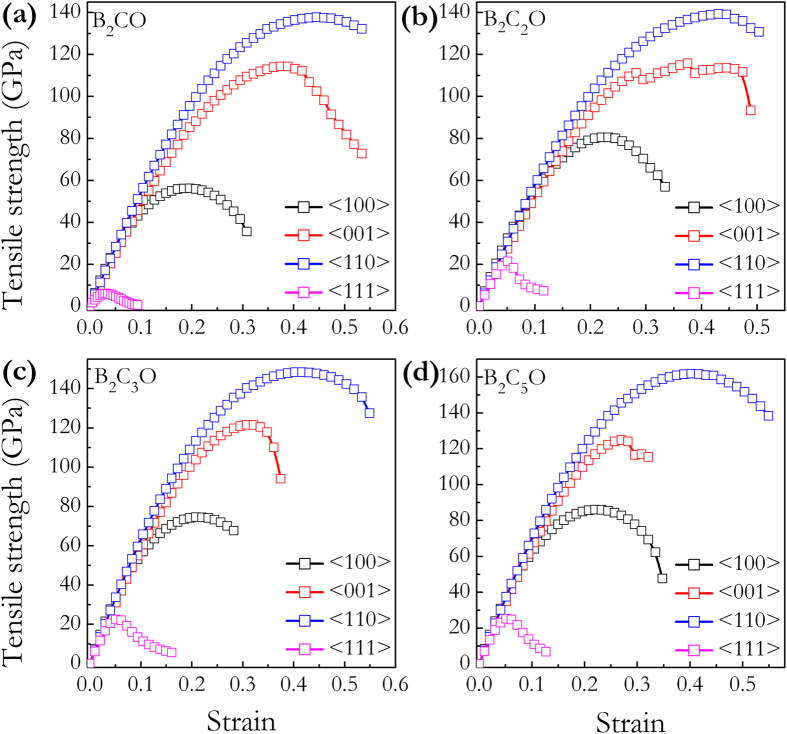
Calculated tensile stress-strain relations. (**a**) tP4-B_2_CO, (**b**) B_2_C_2_O, (**c**) B_2_C_3_O, and (**d**) B_2_C_5_O.

**Figure 8:**
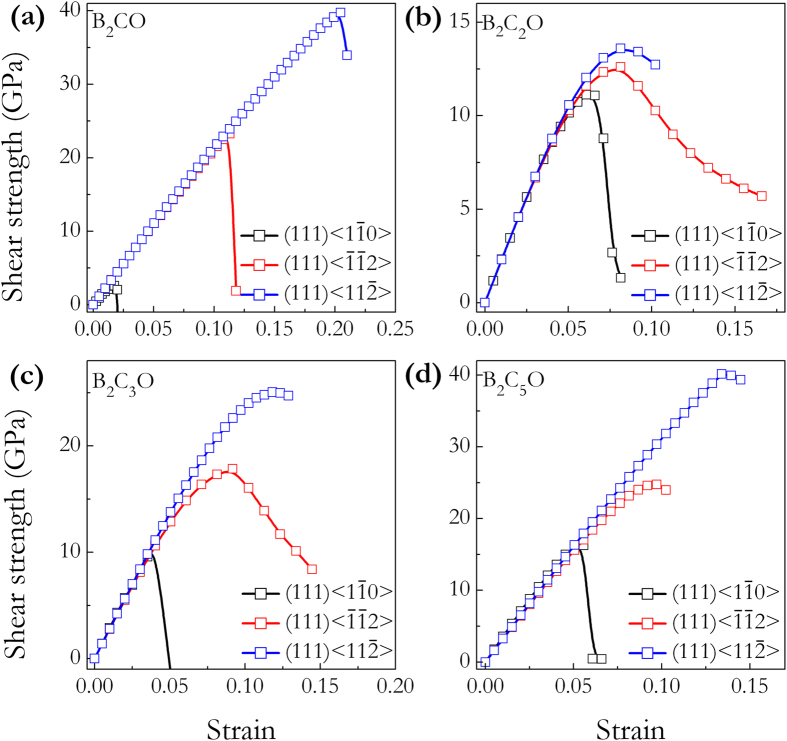
Calculated shear stress-strain relations. (**a**) tP4-B_2_CO, (**b**) B_2_C_2_O, (**c**) B_2_C_3_O, and (**d**) B_2_C_5_O.

**Figure 9:**
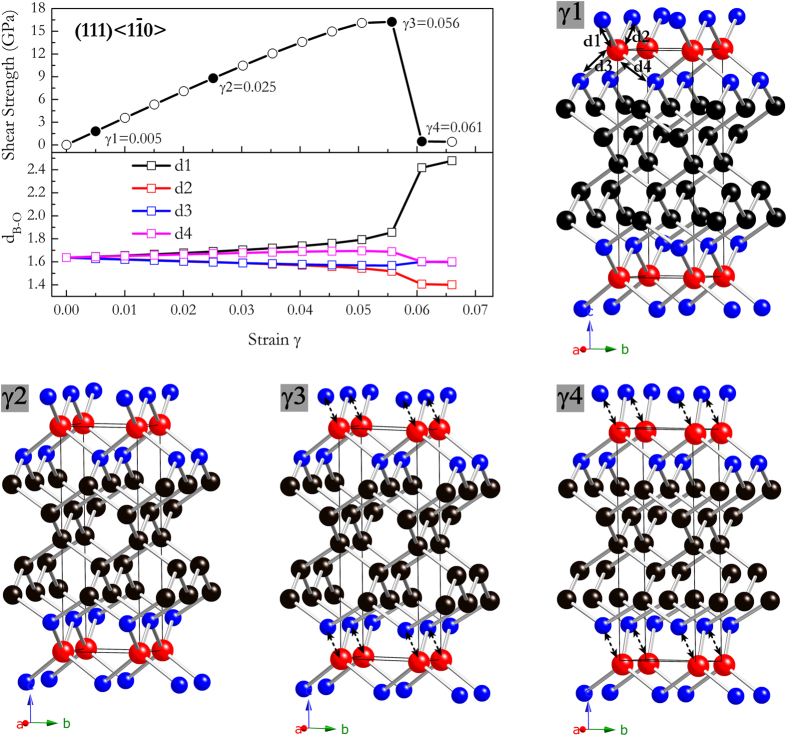
Calculated shear stress-strain relations and the corresponding bond lengths under along (111) 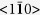 shear strain for B_2_C_5_O. The detailed atomistic structural deformation modes were also shown.

Recently, a well-defined approach^49^,^50^ to understand the structural deformation, strength, and hardness have been extensively applied to strong solids under specified loading strains^51^,^52^,^53^,^54^. It is the ideal strength of a material which is defined as the stress at which a perfect crystal becomes mechanically unstable, that sets an upper bound for material strength. Studies of the strain-stress relations and the underlying atomistic bond-breaking processes can provide critical insights into the atomistic mechanism for the structural deformation and failure modes. Figure 7 presents the calculated strain-stress relations for these four B_2_C_x_O compounds under tensile strains in four principal symmetry crystallographic directions. It can be seen that these B_2_C_x_O compounds have strong stress responses in the < 100 > , < 001 > , and < 110 > directions with peak tensile stresses above 50 GPa. Especially in < 110 > directions, the peak tensile stresses of these compounds (tP4-B_2_CO: 137.4 GPa, B_2_C_2_O: 139.3 GPa, B_2_C_3_O: 148.3 GPa, and B_2_C_5_O: 161.8 GPa) are all higher than those of diamond (126.3 GPa) and *c*-BN (94 GPa) in the same directions^51^. However, compared to diamond (96.3 GPa) and *c*-BN (70.5 GPa) in the < 111 > directions^51^, these B_2_C_x_O possesses much lower peak tensile stresses of 6.1 GPa, 21.3 GPa, 22.4 GPa, and 25.1 GPa, respectively, showing their much weaker tensile resistance or potential superhardness. This result is consistent with their body-diagonal alignment of the weak B-O bonds. One can see that an additional 37.3 wt% of carbon content in B_2_C_5_O over that in B_2_CO leads to 75.7% of enhancement to its ideal tensile strength. However, even with this large increase the ideal tensile strength of B_2_C_5_O is still below those of diamond and *c*-BN. The weakest peak tensile stress occurs in the < 111 > directions, which indicates that under tensile loadings, these B_2_C_x_O compounds would first cleave in the (111) plane. The critical shear stresses in these compounds are then calculated by applying 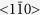

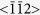
 and 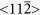 shear deformations in the (111) easy cleavage plane perpendicular to the weakest tensile direction. As shown in Fig. 8, for each B_2_C_x_O compound along (111) 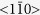 shear direction, the lowest ideal shear strength is determined to be 2.6 GPa, 11.1 GPa, 11.4 GPa, and 16.3 GPa, which is lower than that of the lowest tensile strength, respectively. This means that the failure mode in each B_2_C_x_O phase is dominated by the shear type. Since indentation hardness measurements produce volume and shape changes beneath the indenter, it is expected that shear strength is more closely related to the intrinsic hardness of a material. In fact, it has been shown that the limit of the structural stability is correlated with the maximum shear strength^42^. The present ideal shear strength calculations indicate that these B_2_C_x_O members are likely not to be superhard materials despite their increased C content. Take B_2_C_5_O for example, we next explore the atomistic structural deformation mode along (111) 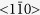 shear direction to understand this result. Figure 9 shows the lengths of the B-O bonds in B_2_C_5_O that connect the shear planes as a function of strain, and the structural snapshots of the unit cell at the critical steps near the bond-breaking points are also plotted. In Fig. 9, the equivalent B-O lengths (1.637 Å at equilibrium state) are stretched and split into four nonequivalence B-O lengths denoted as *d*1, *d*2, *d*3, and *d*4 under shear loadings. The bond lengths of *d*2 and *d*3 decrease nearly synchronously at each strain. On the contrary, *d*1 and *d*4 increase conformably with increasing strains and *d*1 break at the critical shear strain of 0.056, and *d*4 then decreases abruptly along with the breaking of *d*1 bonds. Such a bond-breaking can also be seen from the selected crystal structures (at shear strains of γ1, γ2, γ3, and γ4) before and after shear instability. Therefore, the origin of the lattice instability of B_2_C_5_O under large shear strain at the atomic level during shear deformation can be attributed to the breaking of weak ionic B-O bonds, and this is also the case for other B_2_C_x_O members. According to the results discussed above, the increased number of stronger *sp*^3^ C-C bonds indeed contribute to larger elastic moduli, hardness, and idea strengths to these ternary B_2_C_x_O compounds isoelectronic with diamond. However, because of their significantly lower ideal shear strengths originated from weak B-O bonds than those of superhard diamond and *c*-BN, all these B_2_C_x_O compounds may not be intrinsically superhard.

## Conclusion

In summary, we have systematically explored the crystal structures and properties of ternary B_2_C_x_O (*x* ≥ 2) compounds at ambient conditions by using unbiased structure searching techniques in combination with first-principles calculations. Three novel tetragonal diamond-like structure with group symmetry *I*4_1_/*amd*, *I*-4*m*2, and *P*-4*m*2 for B_2_C_2_O, B_2_C_3_O, and B_2_C_5_O is uncovered, respectively. They are all dynamically stable and can be synthesized at ambient conditions according to the phonon dispersions and formation enthalpies calculations. The elastic anisotropy of each B_2_C_x_O phase has been demonstrated by the distributions of Young’s and shear moduli along different crystal orientations. A good relation between the mechanical behavior and carbon concentration of B_2_C_x_O was established by detailed evaluating the variations of the crystal configurations, electronic structures, and mechanical properties. The present results suggest that the high carbon content benefits that the formation of the covalent *sp*^3^ C-C polyhedral stacking structure and contributes to larger elastic moduli and hardness. The ideal strength calculations, however, indicate that all these B_2_C_x_O compounds may not be intrinsically superhard due to their significantly lower ideal shear strengths than those of diamond and *c*-BN, originated from weak B-O bonds.

## Methods

The crystal structure searches were performed based on a global minimization of energy surfaces merging *ab initio* total-energy calculations as implemented in CALYPSO code^23^,^24^. CALYPSO code was designed to predict stable or metastable crystal structures requiring only chemical compositions of a given compound at given external conditions (e.g., pressure). Here, using the CALYPSO code in combined with Vienna ab initio simulation package (VASP)^55^, variable cell structure searches for each B_2_C_x_O (*x* = 2, 3, 5) compound containing 1–4 f.u. in the simulation cell were systematically performed at ambient pressure. During the structure searchs, the 60% structures of each generation with lower enthalpies were selected to generate the structures for the next generation by Particle Swarm Optimization (PSO) operation, and the other structures in new generation were randomly generated to increase the structural diversity. The underlying structure relaxations and electronic calculations were carried out using density functional theory with all-electron projector-augmented wave (PAW) method^56^ to describe the electron-ion interactions, as implemented in the VASP code. The exchange-correlation functional was treated by the generalized gradient approximation (GGA)^36^ using functional of Perdew Burke Ernzerhof (PBE)^57^. The electronic wave functions were expanded in a plane-wave basis set with cutoff energy of 600 eV, and the Brillouin zone integration was employed using Monkhorst-Pack *k* point meshes^58^ with a grid of 0.03 Å^−1^ for all cases to ensure the total energies converged to be better than ~1 meV/atom. The phonon frequencies were calculated by the direct supercell approach, which uses the forces obtained by the Hellmann-Feynaman theorem calculated from the optimized supercell^59^. The strain-stress method^60^ was used in calculating the single elastic constants. A set of given strains with a finite variation between −0.01 and + 0.01 were applied on the optimized structure and the atomic position was fully optimized. Then, the elastic constants were obtained from the stress of the strained structure. The polycrystalline bulk modulus and shear modulus were thus derived from the Voigt-Reuss-Hill averaging scheme^61^. The quasistatic ideal strength is calculated by incrementally deforming the modeled cell in the direction of the applied strain and controlling the specific strain components, and simultaneously relaxing both the other strain components, as well as the atoms inside the unit cell, at each step.

## Additional Information

**How to cite this article**: Zhang, M. *et al*. Influences of carbon concentration on crystal structures and ideal strengths of B_2_C_x_O compounds in the B-C-O system. *Sci. Rep.* **5**, 15481; doi: 10.1038/srep15481 (2015).

## Supplementary Material

Supplementary Information

## Figures and Tables

**Table 1:** Space group (SG), calculated lattice parameters *a* and *c* (Å), average atomic volume *V*_0_ (Å^3^/atom), density (g/cm^3^), EOS fitted bulk modulus *B*_0_ (GPa), and pressure derivative *B*_0_′ of each B_2_C_x_O.

**Compounds**	**SG**	**Source**	***a***	***c***	***V***_**0**_	***ρ***	***B***_**0**_	***B***_**0**_**′**
tP4-B_2_CO	*P*-4*m*2	Ref. 21	2.623	3.623	6.225	3.309		
		This work	2.660	3.680	6.510	3.170	283	3.699
B_2_C_2_O	*I*4_1_/*amd*	This work	2.647	18.272	6.40	3.198	298	3.742
B_2_C_3_O	*I*-4*m*2	This work	2.613	10.980	6.246	3.263	321	3.746
B_2_C_5_O	*P*-4*m*2	This work	2.588	7.284	6.098	3.325	348	3.633
B_2_O	*I*4_1_/*amd*	Ref. 21	2.688	11.116	6.693	3.11	236	

The results of B_2_O are also listed for comparison.

**Table 2:** The bond length *d* (Å), electron density ( 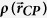 *e*Å^−3^), and Laplacian value 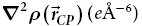 at the bonding critical point of B_2_C_x_O.

**Compounds**	**Bond**	***d***	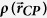	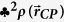
tP4-B_2_CO	B-C	1.572	1.256	−7.924
	B-O	1.665	0.675	6.973
B_2_C_2_O	C-C	1.604	1.357	−7.663
	B-C	1.566	1.318	−7.855
	B-O	1.655	0.682	8.048
B_2_C_3_O	C1-C2	1.585	1.456	−9.346
	B-C2	1.558	1.331	−7.915
	B-O	1.645	0.694	8.645
B_2_C_5_O	C1-C2	1.568	1.549	−11.068
	C1-C3	1.575	1.478	−9.773
	B-C3	1.551	1.346	−8.097
	B-O	1.637	0.703	9.003
Diamond^35^	C-C	1.530	1.60	−15.24

**Table 3:** Calculated single elastic constant *C*_*ij*_ (GPa), Bulk modulus *B* (GPa), Young’s *E* (GPa), Shear modulus *G* (GPa), and Poisson’s ratio *ν* for B_2_C_x_O, together with experimental data for *c*-BN and diamond.

**Compounds**	**Source**	**Method**	***C***_**11**_	***C***_**33**_	***C***_**44**_	***C***_**66**_	***C***_**12**_	***C***_**13**_	***B***	***G***	***E***	***ν***
tP4-B_2_CO	Ref. 21	LDA	736	591	240	254	53	157	311	254		0.18
	This work	LDA	755	593	253	256	42	163	316	265	617	0.17
		GGA	687	550	133	243	44	133	282	197	480	0.22
B_2_C_2_O	This work	GGA	763	590	229	274	15	135	299	264	611	0.16
B_2_C_3_O	This work	GGA	808	664	283	299	32	183	322	302	690	0.14
B_2_C_5_O	This work	GGA	889	740	346	335	30	135	345	351	787	0.12
*c*-BN	Ref. 39	Exp.	820		480		190		400			
Diamond	Ref. 40	Exp.	1076		577		125		442			

**Table 4:** Calculated hardness *H*_*v*_ (GPa) for each B_2_C_x_O compound by using different theoretical models.

**Compounds**	**Source**	***H***_***v***_^**Gao**^	***H***_***v***_^**Chen**^	***H***_***v***_^**Šimůnek**^
tP4-B_2_CO	Ref. 21	50		
	This work	51	40	38
B_2_C_2_O	This work	57	42	43
B_2_C_3_O	This work	62	49	47
B_2_C_5_O	This work	68	60	51
