# Supplementary material for: Influences of carbon concentration on crystal structures and ideal strengths of B2CxO compounds in the B-C-O system
Source: Sci Rep. 2015 Oct 21;5:15481. doi: 10.1038/srep15481 (PMC4614479; doi:10.1038/srep15481)

Supplementary information

Influences of carbon concentration on crystal structures and ideal strengths of B_2C_xO compounds in the B-C-O system

Meiguang Zhang[1*], Haiyan Yan[2], Baobing Zheng[1] & Qun Wei[3]

[1] *College of Physics and Optoelectronic Technology, Nonlinear Research Institute, Baoji University of Arts and Sciences, Baoji 721016, China,* [2] *College of Chemistry and Chemical Engineering, Baoji University of Arts and Sciences, Baoji 721013, China,* [3] *School of Physics and Optoelectronic Engineering, Xidian University, Xi'an* 710071, *China.*

Table S1| Atomic bond lengths (Å) in B_2C_xO compounds at ambient pressure.

Compounds	SG	$d_{C\text{-}C}$		$d_{B\text{-}C}$	$d_{B\text{-}O}$
tP4-B_2CO	*P*-4*m*2			1.572	1.665
B_2C_2O	*I*4$_1$/*amd*	1.604		1.566	1.655
B_2C_3O	*I*-4*m*2	1.585		1.558	1.645
B_2C_5O	*P*-4*m*2	1.568	1.575	1.551	1.637

Figure S1| The calculated phonon dispersion curves for each of B_2C_xO at ambient conditions. (a) *I*4_1/*amd*-B_2C_2O, (b) *I*-4*m*2-B_2C_3O, and (c) *P*-4*m*2-B_2C_5O.

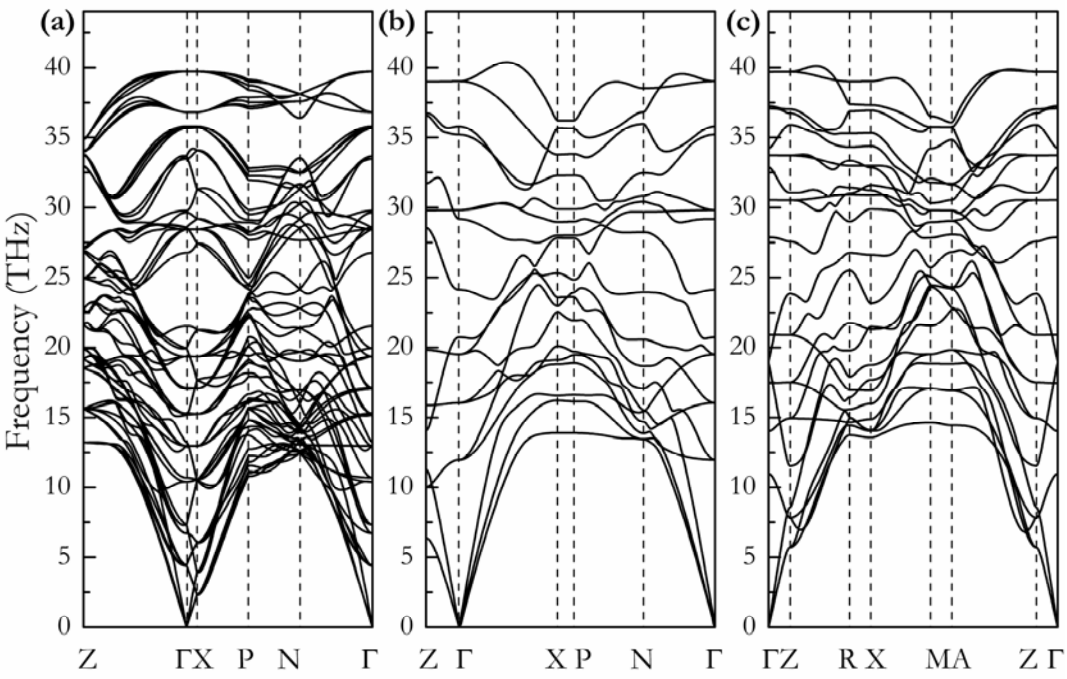

Figure S2| The calculated formation enthalpy for each of B_2C_xO phases as a function of pressure. (a) $\Delta H_{f1} = H_{B_2C_xO} - 2H_B - xH_C - \frac{1}{2}H_{O_2}$ and (b) $\Delta H_{f2} = H_{B_2C_xO} - H_{B_2O} - xH_C$.

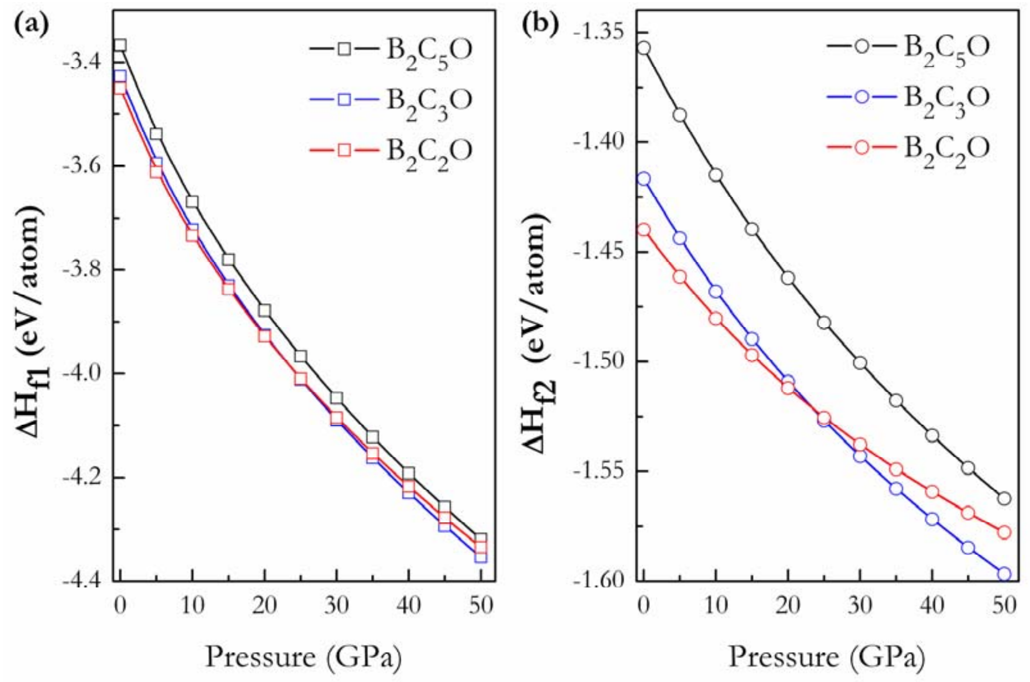

Figure S3 3D ELF distributions of (a) B_2C_3O and (b) B_2C_5O.

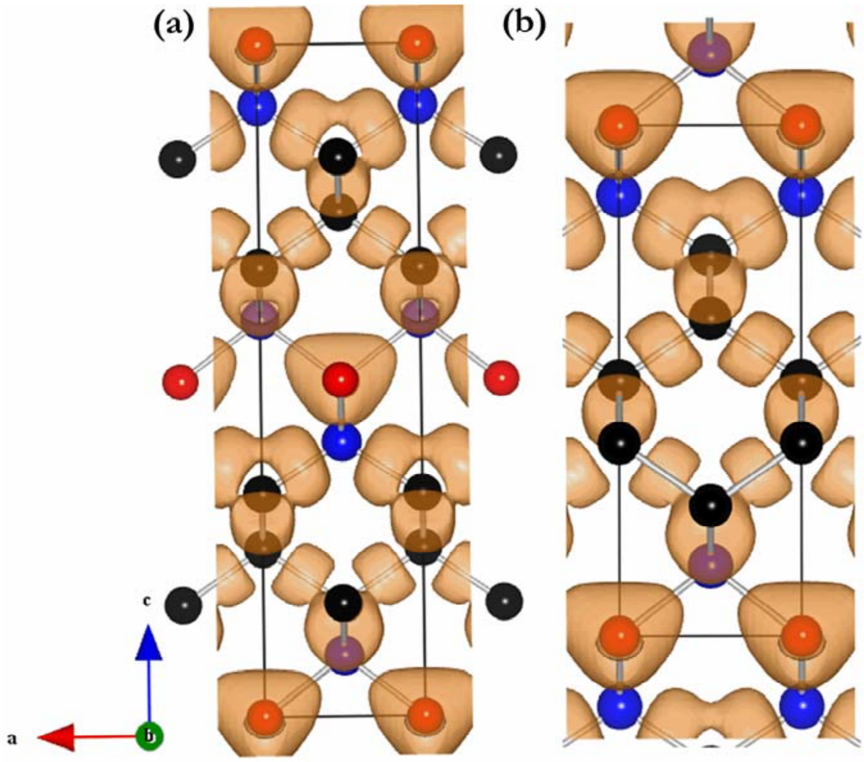

Supplement: Supplementary Information [file srep15481-s1.pdf]
